# 2713. Risk factors and clinical presentation of Herpes Simplex Virus Encephalitis in cancer patients

**DOI:** 10.1093/ofid/ofad500.2324

**Published:** 2023-11-27

**Authors:** Rich Kodama, Mini Kamboj

**Affiliations:** Memorial Sloan Kettering Cancer Center, New York, New York; MSKCC, New York, NY

## Abstract

**Background:**

Herpes Simplex Virus Encephalitis (HSVE) can have lifelong sequelae. Presentation can be nonspecific in cancer patients. Cerebrospinal fluid testing may be falsely negative early in illness [1]. New immune-based or targeted radiation (RT) modalities may change risk, incidence of HSVE in cancer patients.

**Methods:**

Retrospective study at Memorial Sloan Kettering Cancer Center (MSK). Review of records of patients with CSF meningitis/encephalitis (ME) PCR panels from 8/1/2016 to 7/31/2022. All unique patients who received brain RT at MSK during those dates were used to calculate incidence rates.

**Results:**

2749 patients had 5497 CSF analyses with ME panel. Of these, 12 tests from 10 patients showed HSV-1 or -2; 1 patient had relapsing HSVE and 3 positive HSV2 tests each done at least 6 months apart.

Table 1 shows 10 patients with HSVE. 8 had solid tumors, 6 with receipt of chemo- or immunotherapy within 30 days. All solid tumor patients with HSVE had brain RT; 6 received steroids. 1 patient with relapsing HSVE was on immune checkpoint inhibitor (ICI). 4/8 patients with MRIs (Table 2) lacked typical findings; 4/8 patients with EEGs had focal temporal seizure activity. CSF pleocytosis was absent in 4/10 cases. 5 patients had residual deficits. 4 died due to progression of disease.

Incidence of HSVE with brain radiation was 2.6/10,000 patient-years.

**Figure:**
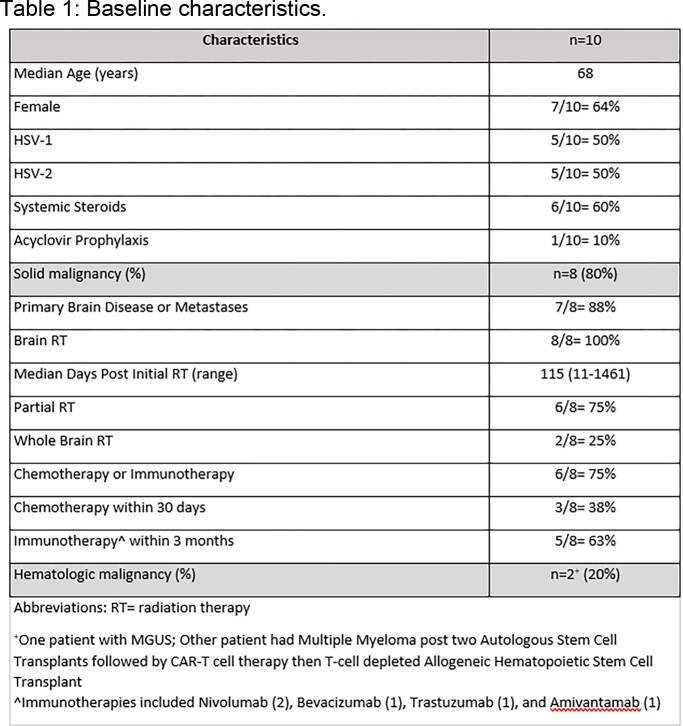

**Figure:**
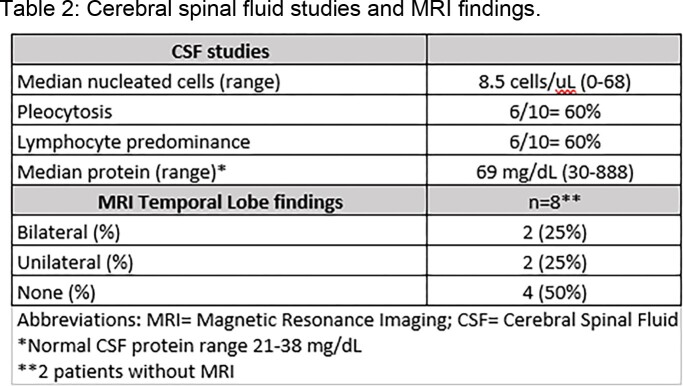

**Conclusion:**

General population incidence of HSVE ranges from 1-4 per million population per year [2]. Those receiving cancer treatment and brain RT have elevated HSVE risk. We found the risk after brain RT is much higher: 2.6/10,000 person-years. Only 1 of 10 patients in our series made a full recovery.

Other key findings include:

*1)* One patient with recurrent HSVE in the setting of nivolumab: ICI combined with brain RT may increase risk of HSV reactivation. Such patients should be considered for high dose antiviral prophylaxis after a first HSVE episode.
*2)* Normal MR imaging and lack of CSF pleocytosis does not preclude HSVE diagnosis in clinically compatible illness.
*3)* No cases occurred among solid tumor patients treated with chemo- and/or immunotherapy only.

Limitations:

*1.* Retrospective design.
*2.* Inclusion of lab confirmed cases only.

**Disclosures:**

**Mini Kamboj, MD** , ASCO: speaker fee|Medscape: speaker fee|MJH life sciences: speaker fee|Regeneron: Advisor/Consultant|Web md: speaker fee

